# The association between cigarette smoking and serum thyroid stimulating hormone, thyroid peroxidase antibodies and thyroglobulin antibodies levels in Chinese residents: A cross-sectional study in 10 cities

**DOI:** 10.1371/journal.pone.0225435

**Published:** 2019-11-25

**Authors:** Yan Zhang, Lixin Shi, Qiao Zhang, Nianchun Peng, Lulu Chen, Xiaolan Lian, Chao Liu, Zhongyan Shan, Bingyin Shi, Nanwei Tong, Shu Wang, Jianping Weng, Jiajun Zhao, Weiping Teng

**Affiliations:** 1 Department of Endocrinology, Affiliated Hospital of Guizhou Medical University, Guiyang, China; 2 Department of Endocrinology, Union Hospital, Tongji Medical College, Huazhong University of Science and Technology, Wuhan, China; 3 Department of Endocrinology, Peking Union Medical College Hospital, Beijing, China; 4 Department of Endocrinology, Jiangsu Province Hospital on Integration of Chinese and Western Medicine, Nanjing, China; 5 Department of Endocrinology and Metabolism, Key Laboratory of Thyroid Diseases in Liaoning Province, The First Hospital of China Medical University, Shenyang, China; 6 Department of Endocrinology, First Affiliated Hospital of Medical College of Xi’an Jiaotong University, Xi’an, China; 7 Department of Endocrinology, West China Hospital, Sichuan University, Chengdu, China; 8 Department of Endocrinology, The Ruijin Hospital of Shanghai Jiaotong University, Shanghai, China; 9 Department of Endocrinology, The Third Affiliated Hospital of Sun Yat-sen University, Guangzhou, China; 10 Department of Endocrinology, The Provincial Hospital Affiliated to Shandong First Medical University, Jinan, China; International University of Health and Welfare, School of Medicine, JAPAN

## Abstract

**Objectives:**

Although several studies have shown that cigarette smoking is associated with thyroid stimulating hormone (TSH), thyroid peroxidase antibody (TPOAb) and thyroglobulin antibody (TgAb), the exact relationship between smoking and thyroid function is controversial. As little is known about the effects of smoking on TSH, TPOAb and TgAb in Chinese residents. This study aimed to evaluate the association between cigarette smoking and TSH, TPOAb and TgAb in ten-city residents of China.

**Study design:**

This was a population-based cross-sectional study.

**Methods:**

In this cross-sectional study, 15,181 subjects from ten major cities of China were investigated. Data regarding demographic characteristics, smoking status and consumption of iodine status were collected using in-person interviews based on a self-designed structured questionnaire. Serum concentrations of TSH, TPOAb and TgAb were measured using electrochemiluminescence immunoassays. Univariate analysis and multivariate linear stepwise regression analyses were used to analyze the data.

**Results:**

The regular smokers had lower concentrations of TSH, TPOAb and TgAb than occasional smokers, former smokers and never smokers. Multivariate analysis demonstrated that regular smoking was associated with the decreased concentrations of TSH (*β* = -0.178), TPOAb (*β* = -0.287) and TGAb (*β* = -0.453) after adjusting other factors. Furthermore, daily smoking number was significantly associated with the decreased level of TSH (*β* = -0.045) and TPOAb(*β* = -0.080), and smoking duration was associated with the decreased TSH level (*β* = -0.030).

**Conclusions:**

Our findings suggest that cigarette smoking is related to a significant decline in the concentrations of TSH, TPOAb and TgAb. In addition, daily smoking number and long-term smoking decrease serum TSH and TPOAb levels. Cigarette smoking plays a significant role in the development of thyroid dysfunction.

## Introduction

Thyroid dysfunction has a significant impact upon the general population[1, 2]. In China, although the prevalence of goiter, Graves’ disease, clinical and subclinical hyperthyroidism have decreased in recent years, the prevalence of subclinical hypothyroidism and thyroid nodules have strikingly increased[3]. It is thought that advanced pharmaceutical engineering and food processing may interfere with medicine and iodine intake, and such interventions may affect or improve euthyroidism. Thyroid dysfunction has been recognized as a global public health problem, which affects 10% of the general population and increases the risk of cardiovascular morbidity and mortality[4]. Given the current trajectory, thyroid disease will become a concerned public health issue and require practical interventions.

Cigarette smoking is the leading cause of avoidable premature mortality, and the number of cigarette smokers exceeds 300 million in China[5, 6]. The prevalence of cigarette smoking in population with the age of ≥ 18 years old has been reported as 62.4% in men and 3.4% in women[7]. Smoking is a risk factor for several diseases, such as cancer, chronic obstructive pulmonary disease, stroke, aneurysm and aortic aneurysms and peripheral vascular disease[8]. Additionally, smoking is regard as an independent risk factor for cardiovascular disease[9]. Studies have reported that smoking is a risk factor for the development of thyroid diseases. For example, several studies have confirmed that in developed countries, smoking can increase thyroid size and nontoxic goiter[10–12]. Cigarette smoking increases the risk and severity of Graves’ disease, Graves’ ophtalmopathy and thyroid multinodularity[12–14].

Thyroid stimulating hormone (TSH), thyroid peroxidase antibody (TPOAb) and thyroglobulin antibody (TgAb) are the most conventional indicators of thyroid dysfunction, including hypothyroidism, hyperthyroidism and autoimmune thyroid diseases. Early diagnosis of thyroid diseases has been clearly shown to improve the prognosis of these diseases, according to the third round of the National Health and Nutrition Examination Survey (NHANES III) in United States, which advise to use the three indexes of TSH, TPOAb and TgAb for consideration of thyroid health[15]. Chemical components of tobacco including nicotine, thiocyanate and benzpyrene have been associated with the inhibition of thyroid hormone synthesis and the promotion of goiter, which can directly or indirectly lead to abnormal thyroid hormone production[16, 17].

Cigarette smoking has multiple, minor effects on thyroid function[18]. The effect of cigarette smoking on thyroid function is associated with age, smoking history, number of cigarettes per day, physical health, environment and iodine status (iodine deficiency or residing in an iodine more than adequate region), and other factors[17, 19, 20]. However, the effect of cigarette on thyroid function is controversial. Some studies have shown that smokers had a marked reduction in thyroid hormone levels compared with non-smokers, while other reports provide inconsistent results[16, 21]. Several population-based studies have reported lower levels of serum TSH in smokers than in non-smokers[22–26], while TSH levels were found to be unaltered in other studies[27–29]. The significance to elucidate the effect of smoking on thyroid function is far-reaching, however, up to date the association between smoking and levels of TSH, TPOAb, and TgAb is far from being clear. Therefore, we conducted this cross-sectional survey across ten cities in China to explore the relationship between cigarette smoking and the concentrations of TSH, TPOAb and TgAb, which may be helpful to further investigation of the impact of smoking on thyroid function.

## Materials and methods

### Ethical statement

This study was approved by the ethics committee of the First Affiliated Hospital of China Medical University (No. 34–2008). Written informed consent was obtained from each participant in accordance with the ethical standards of the responsible committee on human research.

### Study population

The survey was conducted in China and ten cities were chosen according to their historical median urine iodine concentration in school-age children that was representative of iodine-adequate and iodine more than adequate status. They are located in densely populated areas of China, which include Shenyang, Beijing, Jinan, Xi’an, Chengdu, Nanjing, Shanghai, Wuhan, Guiyang and Guangzhou. According to environmental information, six cities (Shenyang, Beijing, Jinan, Shanghai, Chengdu and Guangzhou) were classified as iodine-adequate areas and four cities (Xi’an, Nanjing, Wuhan and Guiyang) were classified as iodine more than adequate areas[3]. The cross-sectional study used multi-stage stratified and cluster sampling methods. The random selection procedure has previously been described[3]. We randomly selected regions from each city, and randomly selected communities from the selected regions. People who met the study requirements were then selected for the survey. Finally, one or two communities include 1500 individuals that were randomly selected in each city, and 15,008 participants agreed and completed the study (S1 Fig). The target population covered participants aged ≥20 of the Han ethnic group and other ethnic minorities, including Hui, Manchu, Korean, Mongol, Miao and Zhuang ethnic groups.

### Study design

This study was a cross-sectional epidemiological survey that included face-to-face interviews and thyroid examinations. Data were collected based on a self-designed structured questionnaire. The questionnaire covered five areas gathered from participant interviews: (1) demographic characteristics such as gender, age, ethnic group, education, occupation, etc.; (2) consumption of iodine status: type of salt used, consumption of kelp and porphyra, and the personal or family history of thyroid diseases (including time of diagnosis and therapy undertaken); (3) smoking status including smoking status, smoking cessation status, tobacco type, years of smoking, age when they began smoking, number of cigarettes per day and passive smoking; (4) physical examination, which considered blood pressure levels, body mass index (BMI) and waistline; and (5) laboratory measurements.

In this study we measured serum TSH, TPOAb and TgAb. Samples of blood were obtained from each subject after an overnight fast. All samples were stored at -20°C and transferred within 1 month of collection to the laboratory in the project center for centralized measurements. TSH, TPOAb and TgAb levels were tested using electrochemiluminescence immunoassays on a Cobas 601 analyzer (Roche Diagnostic, Switzerland). Normal reference ranges were 0.27–4.2 mIU/L for TSH, 0–34 IU/ml for TPOAb and 0–115 IU/ml for TgAb[3]. In those with abnormal serum TSH, free thyroxin (FT4) and free triiodothyronine (FT3) levels was measured. However, the latter was excluded in this study, because FT4 and FT3 had too many missing values and beyond the aim of the study. The functional sensitivity of serum TSH was 0.002 mIU/L. The intra-assay coefficients of variation (CV) of serum TSH, TPOAb and TgAb were 1.1–6.3% and the inter-assay CV values were 1.9–9.5%[3]. And we defined positive antithyroid antibodies as TPOAb >34 IU/ml or TgAb >115 IU/ml[30].

### Implementation

This cross-sectional study lasted over 17 months, from March 2009 to August 2010 across ten cities of China. Investigators and physicians were recruited in each survey region before the implementation of this study. To ensure objectivity and authenticity, all participating investigators and physicians performing physical examinations received strict and standardized professional training before the implementation of the project. A total of 71 physicians and surveyors participated in the on-site survey. All survey subjects were selected strictly according to the random sampling method. During the survey, a supervisor in each region oversaw the surveyors and verified their work; and 10% of the questionnaires completed each day were randomly inspected for completeness, accuracy and standardization. After the whole investigation was completed, the questionnaires were entered twice into computers by two different inputters blinded to the study goals. Based on the questionnaire, the participants in this study were divided into never smoker, former smoker, occasional smoker, and regular smoker groups. People who had never smoked were defined as never smokers. People who smoked previously but had quit currently were defined as former smokers. Current smokers who smoked less than 1 cigarette per day were occasional smokers, and those who smoked more than 1 cigarette per day were regular smokers.

### Statistical analysis

A descriptive analysis was performed to describe demographic characteristics of study participants. Categorical variables were reported as numbers and percentages. Quantitative data that were normally distributed were expressed as means and standard deviations while non-normally distributed data were expressed as medians and quartiles (P_25_~P_75_). Cigarette smoking status was described as ratio, and the concentrations of TSH, TPOAb and TgAb were presented as medians and quartiles (P_25_~P_75_). Differences of demographic characteristics among smoke status groups were evaluated using ANOVA and chi-squared (*χ*^2^) test. A Mann-Whitney test or a Kruskal-Wallis test was used to compare the differences of TSH, TPOAb and TgAb between groups. Since TSH, TPOAb and TgAb as dependent variables had no normal distribution, we applied Ln TSH, Ln TPOAb and Ln TgAb for multivariable analysis. Multivariable linear regression analyses were used to evaluate the associations of cigarette smoking with Ln TSH, Ln TPOAb and Ln TgAb. For all outcomes, significant variables (*p*<0.05) in univariate analyses were entered into the multivariate models and retained in the final models if *p*< 0.05. All statistical analyses were performed using SPSS 17.0 (SPSS Inc., Chicago, IL, USA).

## Results

### The demographic characteristics of participants

The study included 13,512 subjects with complete data enrolled at this epidemiological survey (i.e. effective response rate of 90.0%). Of the 13,512 subjects surveyed, 7,761 (57.4%) participants were from iodine-adequate areas and 5,751 (42.6%) lived in iodine more than adequate areas. Among them, 115 were defined as former smokers (61 males, 54 females), 396 were occasional smokers (276 males, 120 females), 3,127 were regular smokers (2,938 males, 189 females), and 9,874 were never smokers (2,454 males, 7,420 females).

The characteristics of the entire sample among smokers and non-smokers are described and compared in Table 1. The mean age (± standard deviation; SD) was 45.61 (±14.92) for the entire population. There were significant differences between these four groups for age (*p*<0.001), gender (*p*<0.001), frequency of postmenopausal women (*p* = 0.005), frequency of abnormal childbearing (*p*<0.001), BMI (*p*<0.001), waistline (*p*<0.001), hypertension (*p*<0.001) and family history of thyroid diseases (*p*<0.001). However, no significant difference was found among different smoking status groups for Han ethnicity (*p* = 0.618). Other demographic characteristics of study participants are displayed in S1 Table.

**Table 1:** Demographic characteristics of study participants in each group.

Variables	Never smokers	Former smokers	Occasional smokers	Regular smokers	*p*
**Age**	45.84±15.31	51.36±14.11^a^	42.42±13.59^a^^b^	45.06±13.76^a^^b^^c^	<0.001
**Males, n (%)**	2454 (24.9%)	61 (53.0%)	276 (69.7%)	2938 (94.0%)	<0.001
**Postmenopausal women, n (%)**	2834 (38.2%)	33 (61.1%)	45 (37.5%)	79 (41.8%)	0.005
**Abnormal childbearing, n (%)**	1966 (26.5%)	26 (48.1%)	32 (26.7%)	74 (39.2%)	<0.001
**Han Chinese**	9581 (97.0%)	112 (97.4%)	383 (96.7%)	3020 (96.6%)	0.618
**BMI (kg/m**^**2**^**)**	23.59±3.46	24.66±3.23^a^	24.27±3.34^a^	24.82±3.49^a^^c^	<0.001
**Waistline (cm)**	79.32±9.87	84.36±9.57^a^	82.98±9.15^a^	85.78±9.48^a^^c^	<0.001
**Hypertension, n (%)**	2163 (22.0%)	42 (36.5%)	90 (22.8%)	914 (29.3%)	<0.001
**Family history of thyroid diseases, n (%)**	655 (6.6%)	11 (9.6%)	24 (6.1%)	124 (4.0%)	<0.001

^a^*p* <0.05 *vs*. never smokers

^b^*p* <0.05 *vs*. former smokers

^c^*p* <0.05 *vs*. occasional smokers.

### Smoking and iodine uptake situation

The percentage of people with current smoking was 26.1%, the percentage of people who occasionally smoked was 2.9%, and the percentage of people who regularly smoked was 23.1%. Among male participants, the percentage of regular smoking was as high as 51.3% (S2 Fig). The percentage of subjects who were exposed to second-hand smoke was 30.7%. Among those who smoked, their mean age of onset smoking was 22.6±7.0 years, the smoke duration was 20.5±12.5 years (S3 Fig).

As shown in S2 Table, 98.4% of participants had iodized salt intake, there were 1779 (13.2%) subjects without kelp and porphyra consumption at ordinary times, and only 3.1% had iodine drugs use. Of the three common indexes of iodine uptake, significant differences were found between smoking status groups for iodized salt (*p* = 0.019), kelp and porphyra consumption (*p* = 0.001) and iodine drugs use (*p* = 0.003). The results suggested that smokers had more iodine uptake than non-smokers.

### The association between cigarette smoking and TSH, TPOAb, TgAb

Table 2 displays a summary of the concentrations of TSH, TPOAb and TgAb among those with different smoking behaviors. Kruskal-Wallis tests were used to compare the difference, and significant differences were found among different smoking status groups for TSH (*p*<0.001), TPOAb (*p* = 0.017) and TgAb (*p*<0.001). However, no significant differences were found between dry tobacco and cigarette groups for TSH (*p* = 0.679), TPOAb (*p* = 0.220) and TgAb (*p*<0.728).

**Table 2:** Concentrations of TSH, TPOAb and TgAb among different smoking status groups (Median, P_25_~P_75_).

	TSH (mIU/L)	TPOAb (IU/ml)	TgAb (IU/ml)
**Smoking status**	<0.001*	0.017*	<0.001*
Never smokers	2.62 (1.76~3.84)	8.73 (5.79~14.53)	10.00 (10.00~21.70)
Former smokers	2.92 (1.96~3.82)	8.68 (6.06~12.53)	10.00 (10.00~12.73)
Occasional smokers	2.29 (1.64~3.47)	8.45 (5.35~12.47)	10.00 (10.00~12.26)
Regular smokers	2.13 (1.49~3.03)	8.19 (5.06~13.36)	10.00 (10.00~11.11)
**Type of tobacco**	0.679^#^	0.220^#^	0.728^#^
Dry tobacco	2.06 (1.34~3.47)	7.88 (5.32~12.60)	10.00 (10.00~11.05)
Cigarette	2.13 (1.50~3.06)	8.66 (5.97~12.48)	10.00 (10.00~11.28)
**Smoking duration**	<0.001*	<0.001*	0.654*
≤5 years	2.32 (1.60~3.41)	8.99 (6.17~13.26)	10.00 (10.00~11.16)
6~10 years	2.32 (1.64~3.27)	8.79 (6.19~12.58)	10.00 (10.00~11.77)
1~20 years	2.11 (1.52~2.99)	8.51 (5.91~12.08)	10.00 (10.00~11.32)
>20 years	2.05 (1.44~3.05)	7.27 (5.00~10.94)	10.00 (10.00~11.24)
**Daily smoking amount**	<0.001*	<0.001*	0.911*
1~10 cigarettes per day	2.24 (1.55~3.23)	9.33 (7.16~13.71)	10.00 (10.00~11.16)
11~20 cigarettes per day	2.07 (1.47~3.03)	8.82 (6.23~13.05)	10.00 (10.00~11.44)
≥21 cigarettes per day	1.96 (1.42~2.87)	8.32 (5.61~11.94)	10.00 (10.00~11.34)
**Passive smoking**	<0.001^#^	0.810^#^	0.001^#^
Yes	2.55 (1.70~3.73)	8.70 (5.76~14.19)	10.00 (10.00~17.34)
No	2.38 (1.63~3.41)	8.62 (5.81~13.59)	10.00 (10.00~14.61)

*The *p* value of Kruskal-Wallis tests

^#^the *p* value of Mann-Whitney tests.

**Fig 1:**
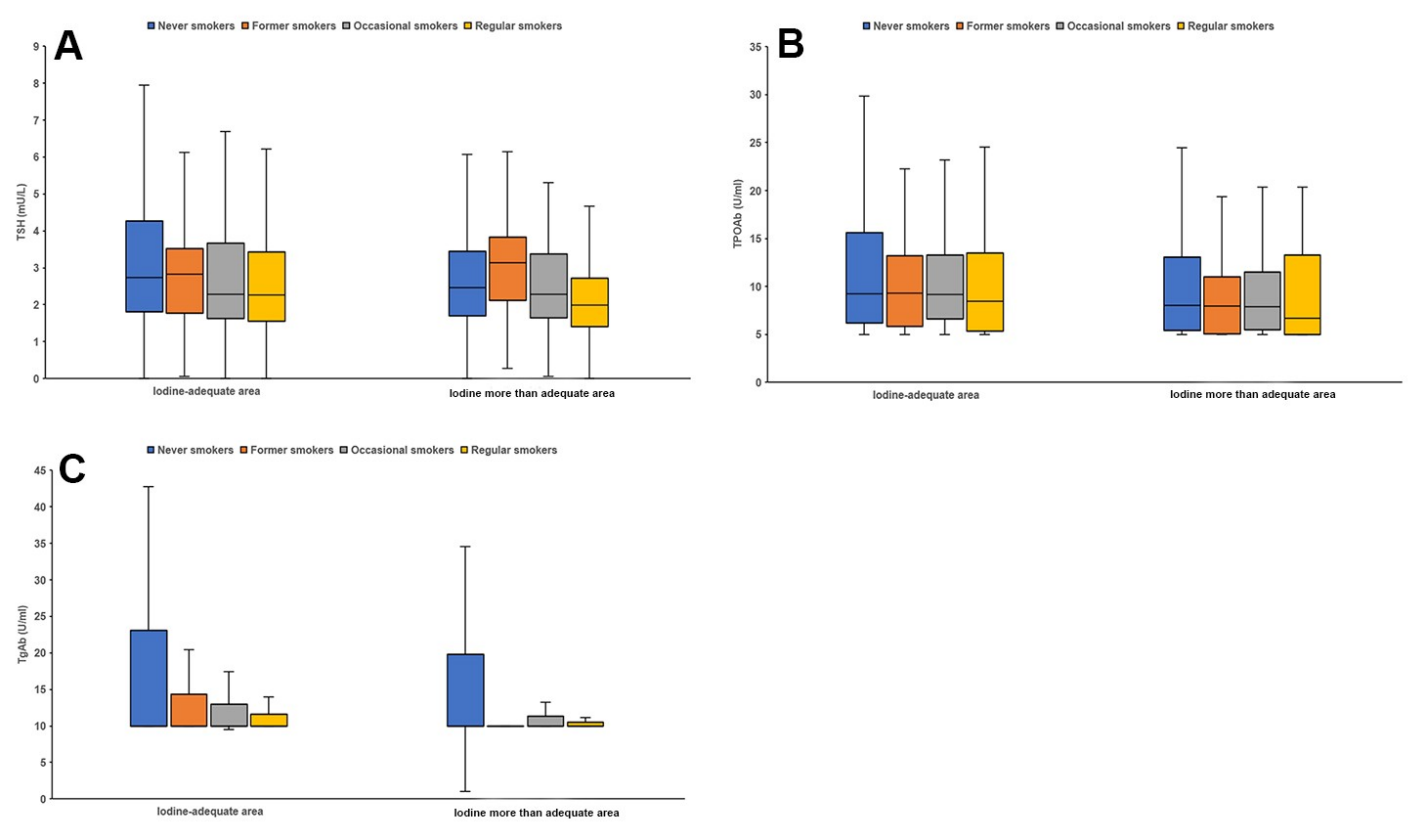
**Box plot of TSH (A), TPOAb (B) and TgAb (C) levels among different smoking status groups between iodine-adequate area and iodine more than adequate area.** The data were expressed as minimum, P_25_, median, P_75_, maximum.

Among smokers, TSH and TPOAb were significantly affected by smoking duration and daily smoking amount. The levels of TSH and TPOAb showed declining trend with the increase of smoking years and the daily smoking amount, while no effect of smoking duration and daily smoking amount on TgAb was found. The levels of TSH and TgAb in passive smokers were significantly higher than that in non-passive smokers. However, no significant differences were found between passive smokers and non-passive smokers for TPOAb. TSH, TPOAb and TgAb levels among different smoking status groups between iodine-adequate area and iodine more than adequate area are presented in Fig 1. Kruskal-Wallis tests showed that the levels of TSH, TPOAb and TgAb were significantly different among different smoking status groups (*p*<0.05). In addition, the proportion of TPOAb positivity and TgAb positivity among different smoking status groups is shown in Table 3. The proportion of TPOAb and TgAb was decreased for smokers than never smokers.

**Table 3:** The proportion of TPOAb positivity and TgAb positivity among different smoking status groups.

Smoking status	TPOAb	TgAb
**Total**	1510 (11.2%)	1669 (12.4%)
**Never smokers**	1100 (11.1%)	1231 (12.5%)
**Former smokers**	9 (7.8%)	10 (8.7%)
**Occasional smokers**	32 (8.1%)	43 (10.9%)
**Regular smokers**	369 (11.8%)	385 (12.3%)

### Multivariate analysis

Since TSH, TPOAb and TgAb as dependent variables had not normal distribution by normality tests, we applied Ln TSH, Ln TPOAb and Ln TgAb for multiple linear regression. Multivariate analysis of demographic characteristics, iodine uptake situation, cigarette smoking, family history of thyroid diseases and physical examination as independent factors associated with Ln TSH (Table 4). Regression analysis suggested that regular smoking, smoking duration and daily smoking amount were related to a decline in the concentration of Ln TSH after adjusting for other variables (*β* = -0.178, -0.030 and -0.048, respectively). Of these factors, regular smoking was strongly associated with Ln TSH (standardized *β* = -0.086) and monthly income was the least influential factor on Ln TSH (standardized *β* = -0.032).

**Table 4:** Multivariate analysis of factors related to Ln TSH.

Variable	*β*	*S*.*E*.	Standardized *β*	*t*	*p*
**Gender**	0.654	0.113	0.068	5.77	<0.001
**Iodine status**	-0.525	0.089	-0.055	-5.89	<0.001
**Age**	0.015	0.003	0.047	4.94	<0.001
**Family history of TD**	0.881	0.188	0.044	4.68	<0.001
**Iodine drugs use**	1.013	0.254	0.037	3.99	<0.001
**Monthly income**	-0.139	0.042	-0.032	-3.30	0.001
**Smoking status**					
**Former smokers**	0.076	0.082	0.008	0.93	0.353
**Occasional smokers**	-0.064	0.045	-0.012	-1.45	0.148
**Regular smokers**	-0.178	0.018	-0.086	-9.99	<0.001
**Smoking duration**	-0.030	0.011	-0.049	-2.72	0.007
**Daily smoking amount**	-0.045	0.021	-0.039	-2.17	0.030
**Constant**	2.630	0.310	-	8.49	<0.001

Ln, natural logarithm. TSH, thyroid stimulating hormone. *S*.*E*., Standard error. TD, thyroid diseases.

Gender: male or female, reference: male.

Iodine status: iodine-adequate area or iodine more than adequate area, reference: iodine-adequate area.

Age: continuous variable.

Family history of TD, reference: those with no family history of TD.

Iodine drugs use, reference: those with no taking iodine drugs.

Monthly income, ordinal categorical variable.

Smoking status, reference: never smokers.

Smoking duration, ordinal categorical variable.

Daily smoking amount, ordinal categorical variable.

Parameter estimates and test results of the multifactorial linear regression model for TPOAb are presented in Table 5. Multivariable analysis showed that former smoking (*β* = -0.370), occasional smoking(*β* = -0.263), regular smoking (*β* = -0.287) and daily smoking amount (*β* = -0.080) were significantly associated with a decline Ln TPOAb after adjusting for other factors. According to the standardized regression coefficients of each variable, regular smoking and gender exhibited the higher values (Standardized *β* = -0.108 and 0.105), which indicated that regular smoking and gender were the important factors that affected the concentration of TPOAb.

**Table 5:** Multivariate analysis of factors related to Ln TPOAb.

Variable	*β*	*S*.*E*.	Standardized *β*	*t*	*p*
**Gender**	22.940	1.873	0.105	12.25	<0.001
**Smoking status**					
**Former smokers**	-0.370	0.122	-0.030	-3.04	0.002
**Occasional smokers**	-0.263	0.065	-0.040	-4.02	<0.001
**Regular smokers**	-0.287	0.056	-0.108	-5.14	<0.001
**Daily smoking amount**	-0.080	0.013	-0.055	-6.38	<0.001
**Family history of TD**	26.279	3.901	0.058	6.74	<0.001
**Age**	0.338	0.062	0.047	5.46	<0.001
**Iodine status**	-8.116	1.879	-0.037	-4.32	<0.001
**Kelp and porphyra consumption**	-5.563	1.840	-0.026	-3.02	0.003
**Iodized salt**	-15.029	7.287	-0.018	-2.06	0.039
**Constant**	17.973	8.953	-	2.01	0.045

Ln, natural logarithm. TPOAb, thyroid peroxidase antibody. *S*.*E*., Standard error. TD, thyroid diseases.

Gender: male or female, reference: male.

Smoking status, reference: never smokers.

Daily smoking amount, ordinal categorical variable.

Family history of TD, reference: those with no family history of TD.

Age: continuous variable.

Iodine status: iodine-adequate area or iodine-rich area, reference: iodine-adequate area.

Kelp and porphyra consumption, reference: those with no taking kelp and porphyra.

Iodized salt, reference: no iodized salt.

Table 6 shows the result of multivariate analysis of factors related to Ln TgAb. Former smoking (*β* = -0.268), occasional smoking(*β* = -0.316), regular smoking (*β* = -0.453) and passive smoking (*β* = 0.063) were significantly associated with a decline in Ln TgAb even after adjustment with other factors, and regular smoking was the strongest influence factor to affect the concentration of TgAb (Standardized *β* = -0.149).

**Table 6:** Multivariate analysis of factors related to Ln TgAb.

Variable	*β*	*S*.*E*.	Standardized *β*	*t*	*p*
**Gender**	125.712	19.292	0.115	6.52	<0.001
**Family history of TD**	68.073	23.922	0.050	2.85	0.004
**Age**	0.985	0.340	0.051	2.90	0.004
**Smoking status**					
**Former smokers**	-0.268	0.119	-0.019	-2.25	0.024
**Occasional smokers**	-0.316	0.065	-0.042	-4.87	<0.001
**Regular smokers**	-0.453	0.026	-0.149	-17.36	<0.001
**Passive smoking**	0.063	0.024	0.023	2.63	0.009
**Constant**	-28.271	48.632	-	-0.58	0.561

Ln, natural logarithm. TgAb, antithyroglobulin antibody. *S*.*E*., Standard error. TD, thyroid diseases.

Gender: male or female, reference: male.

Family history of TD, reference: those with no family history of TD.

Age: continuous variable.

Smoking status, reference: never smokers.

Passive smoking: yes or no, reference: no.

## Discussion

This cross-sectional study is the largest scale survey to date on the correlation of cigarette smoking with thyroid function in China. In this study we have found that cigarette smoking is associated with lower serum TSH, TPOAb and TgAb values, which may lead to lower risk of hypothyroidism and possibly lower frequency of thyroid autoimmune diseases such as Hashimoto's thyroiditis. This finding has important significance since early detection and intervention is necessary in high-risk populations that are known to smoke.

This study has identified that smoking is associated with a decreased serum concentration of TSH after adjusting for gender, age, iodine-intake status, family history of thyroid diseases and other variables. Slightly reduced serum TSH concentrations in smokers have been found in many previous and a few recent studies[14, 17, 20, 22–26, 31–34]. However, the influence of smoking on TSH is controversial. Some studies have reported no association between serum TSH levels and smoking[16, 27–29]. The study also shows that the TSH level decreases with the increase of the number of smokers per day and the years of smoking. In addition, although passive smoking was not included in the multivariate model, univariate analysis revealed that TSH levels in passive smokers were significantly higher than those in non-passive smokers. Some epidemiological studies have reported lesser TSH levels not in all passive smokers compared with non-smokers[35–37]. The mechanism of passive smoking on TSH remains unclear. The reason for a lower serum TSH level among smokers cannot be identified from our study. One possible mechanism is that cyanide is transformed into thiocyanate in the body during cigarette smoking, and thiocyanate can concomitantly lead to reduced iodine adsorption and increased iodide efflux, which further enhances the promoting effect of sympathetic stimulation or increases autoimmune thyroid function, which consequently regulates and increases the synthesis of thyroid hormones[33, 38]. Thyroid hormones reduce the level of TSH through thyroxin feedback inhibition, thus maintaining normal metabolic and organ functions in the smoking population.

TPOAb and TgAb are indicators of autoimmune thyroid disease. However, the association between TPOAb/TgAb and cigarette smoking is controversial. One study from Korea found that smoking status is not associated with the presence of TPOAb, and smoking has a direct effect on thyroid function which is not mediated by autoimmune processes[39]. As reported in the third NHANES, the impact of smoking on TPOAb is related to urinary iodine and racial-ethnic differences. The protective effect of smoking on TPOAb prevalence is only observed in subjects with normal urinary iodine but not in subjects with less or great levels, and the protective effect was only significant in the non-Hispanic white cohort but not in non-Hispanic black, Mexican-American, or other cohorts[34]. One study from China revealed that long-term smoking could increase the prevalence of thyroid autoantibodies in a population with mildly deficient iodine intake[40]. But there are extensive studies showing decreases in risk of positive TPOAb and TgAb associated with smoking[20, 41–44]. In NHANES, fewer smokers had TPOAb and/ or TgAb compared with non-smokers[34]. The percentage of nonsmokers with positive TPOAb and TgAb was significantly higher than that of smokers, i.e. smoking was negatively associated with the presence of thyroid antibodies (TPOAb/TgAb)[45]. Another study reported that TPOAb were significantly lower among smokers but no association between smoking and the other antibodies was observed[46]. Our study has identified that smoking appears to influence not only the lower serum concentration of TSH level but also the concentration of TPOA and TgAb, which suggests that smokers may be less likely to have positive TPOAb/ TgAb than never smokers. In addition, the dose of smoking was associated with a decreased level of TPOAb, but not with TgAb in our multivariate models. This study also revealed that TgAb levels in passive smokers were significantly higher than those in non-passive smokers. The lower level of autoantibody in smokers may be restricted to Hashimoto's thyroiditis[47]. While smoking is a risk factor for Graves' disease[48]. Reduced risk of TPOAb/ TgAb positivity in smokers may be due to smoke effects on cell mediated immunity[49, 50]. Smoking can inhibit natural killer cell activity, increase CD3+ and CD4+ T cell numbers, and reduce CD8+ T cell numbers[50]. CD8+ T-cells participate in autoimmune processes and recognize TPO- and TG-antigens, while natural killer cell and CD4+ T-cells are also involved in the development of thyroid dysfunction[51, 52]. Other studies have noted that the reason for reducing risk of TPOAb/ TgAb positivity in smokers is the interference of smoke in iodide transport and organification, decreased TSH secretion or smoke effects on decreased hormonal and inhibition of prostaglandin synthesis[53, 54]. The protective effect of smoking on TPOAb and TgAb is observed in our study. However, since this study is a cross-sectional study, the causality between smoking and TPOAb/ TgAb cannot be confirmed. Prospective studies are needed in the further.

The limitations of our study are as follows: FT3 and FT4 are two important direct indicators of thyroid function. However, FT3 and FT3 levels were only measured if TSH was outside the reference range. Therefore, we did not evaluate the relationship between smoking and both FT3 and FT4 in our study. The accuracy of using a questionnaire to define smoking status groups was less than NHANESIII[34], which examined serum cotinine levels. Lastly, this cross-sectional study does not permit to assess changes of serum TSH, TPOAb and TgAb over time in the study participants.

In summary, cigarette smoking is related to a significant decline in the concentrations of TSH, TPOAb and TgAb after adjusting for other variable, especially in regular smokers. In addition, the number of cigarettes smoked per day and long-term smoking could decrease serum TSH and TPOAb levels. This finding suggests that smoking has a crucial environmental effect and may play a significant role in the development of thyroid dysfunction.

## Supporting information

S1 Table Other demographic characteristics of study participants. (DOCX)

S2 Table Iodine uptake situation of study participants in each group. (DOCX)

S1 Fig Selection procedures of this cross-sectional epidemiological survey data. (TIF)

S2 Fig The percentage of different smoking status in ten-city residents in China. (TIF)

S3 Fig The age of onset smoking and smoke duration of the smokers. (TIF)
