# Supplementary material for: The association between cigarette smoking and serum thyroid stimulating hormone, thyroid peroxidase antibodies and thyroglobulin antibodies levels in Chinese residents: A cross-sectional study in 10 cities
Source: PLoS One. 2019 Nov 25;14(11):e0225435. doi: 10.1371/journal.pone.0225435 (PMC6876836; doi:10.1371/journal.pone.0225435)
Supplement: S1 Table — (DOCX) [file pone.0225435.s001.docx]

**S1 Table. Other demographic characteristics of study participants (n=13,512).**

| **Category** | **Answer** | **n** | **Proportion (%)** |
| --- | --- | --- | --- |
| **Educational status** | Primary or less | 1208 | 8.9 |
|  | Middle | 3023 | 22.4 |
|  | High | 3995 | 29.6 |
|  | College | 5032 | 37.2 |
|  | Post-graduate | 254 | 1.9 |
| **Monthly income (RMB)** | <1000 | 3231 | 23.9 |
|  | 1000-1999 | 5566 | 41.2 |
|  | 2000-2999 | 2833 | 21.0 |
|  | 3000-9999 | 1807 | 13.4 |
|  | ≥10000 | 75 | 0.5 |
| **Occupation** | Leading cadres | 1854 | 13.7 |
|  | Professionals | 3568 | 26.4 |
|  | Production staff | 3428 | 25.4 |
|  | Soldiers | 38 | 0.3 |
|  | Students | 162 | 1.2 |
|  | Housewife or retirees | 4064 | 30.1 |
|  | Missing | 398 | 2.9 |
