# Supplementary material for: The association between cigarette smoking and serum thyroid stimulating hormone, thyroid peroxidase antibodies and thyroglobulin antibodies levels in Chinese residents: A cross-sectional study in 10 cities
Source: PLoS One. 2019 Nov 25;14(11):e0225435. doi: 10.1371/journal.pone.0225435 (PMC6876836; doi:10.1371/journal.pone.0225435)
Supplement: S2 Table — (DOCX) [file pone.0225435.s002.docx]

**S2 Table. Iodine uptake situation of study participants in each group.**

| **Smoke status** | **Iodized salt** | **Kelp and porphyra consumption** | | **Iodine drugs use** |
| --- | --- | --- | --- | --- |
|  |  | Occasional | Regular |  |
| **Total** | 13290 (98.4%) | 10058 (74.4%) | 1675 (12.4%) | 414 (3.1%) |
| **Never smokers** | 9692 (98.2%) | 7376 (74.7%) | 1252 (12.7%) | 333 (3.4%) |
| **Former smokers** | 113 (98.3%) | 81 (70.4%) | 13 (11.3%) | 4 (3.5%) |
| **Occasional smokers** | 390 (98.5%) | 306 (77.3%) | 52 (13.1%) | 13 (3.3%) |
| **Regular smokers** | 3095 (99.0%) | 2295 (73.4%) | 358 (11.4%) | 64 (2.0%) |
| ***p* value** | 0.019 | 0.001 | | 0.003 |
