# Supplementary figures and images for: The association between cigarette smoking and serum thyroid stimulating hormone, thyroid peroxidase antibodies and thyroglobulin antibodies levels in Chinese residents: A cross-sectional study in 10 cities
Source: PLoS One. 2019 Nov 25;14(11):e0225435. doi: 10.1371/journal.pone.0225435 (PMC6876836; doi:10.1371/journal.pone.0225435)

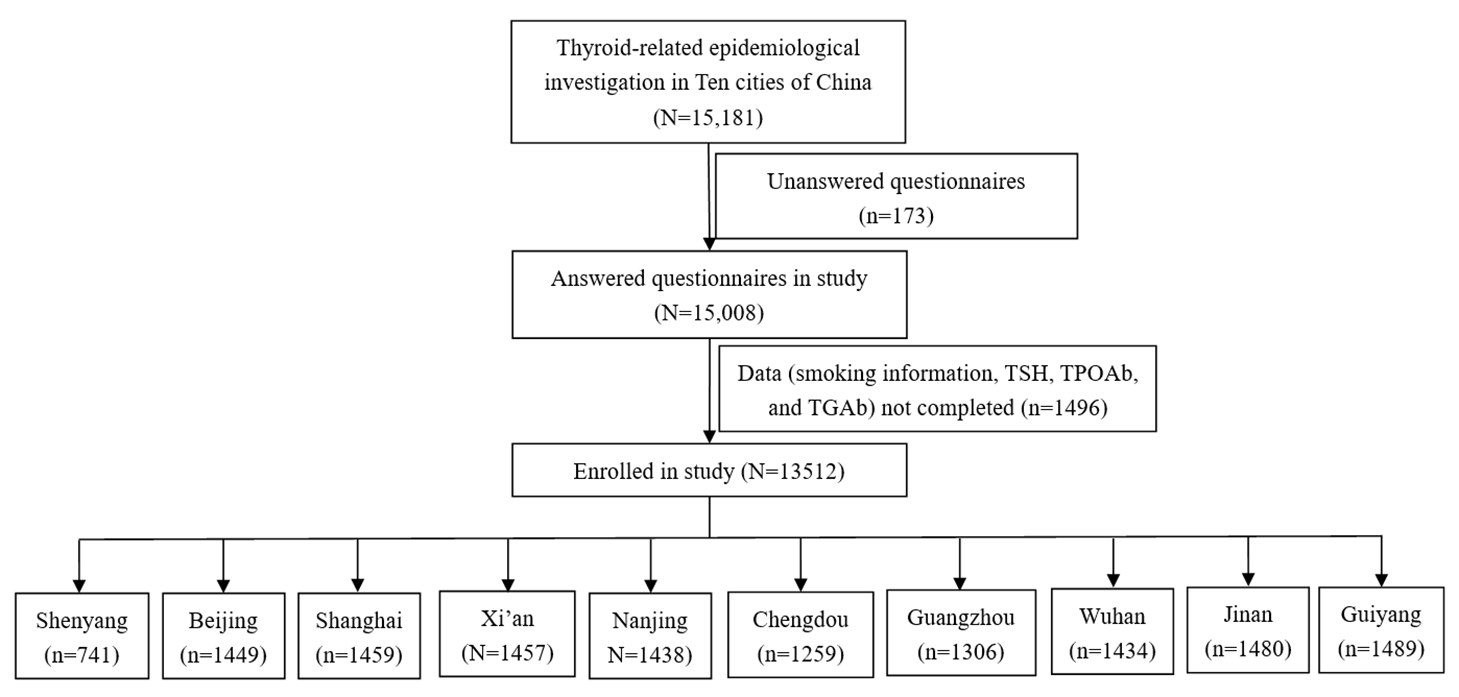

Supplement: S1 Fig — (TIF) [file pone.0225435.s003.tif]

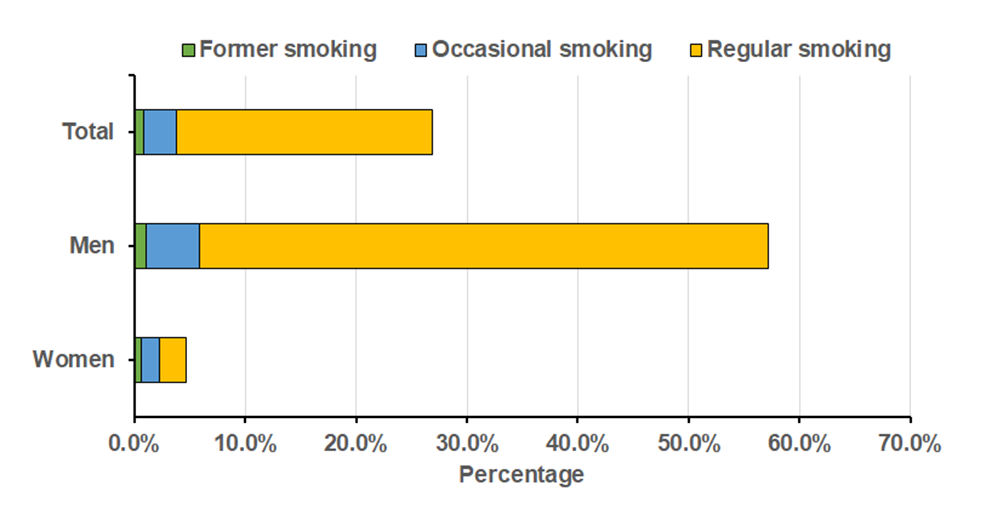

Supplement: S2 Fig — (TIF) [file pone.0225435.s004.tif]

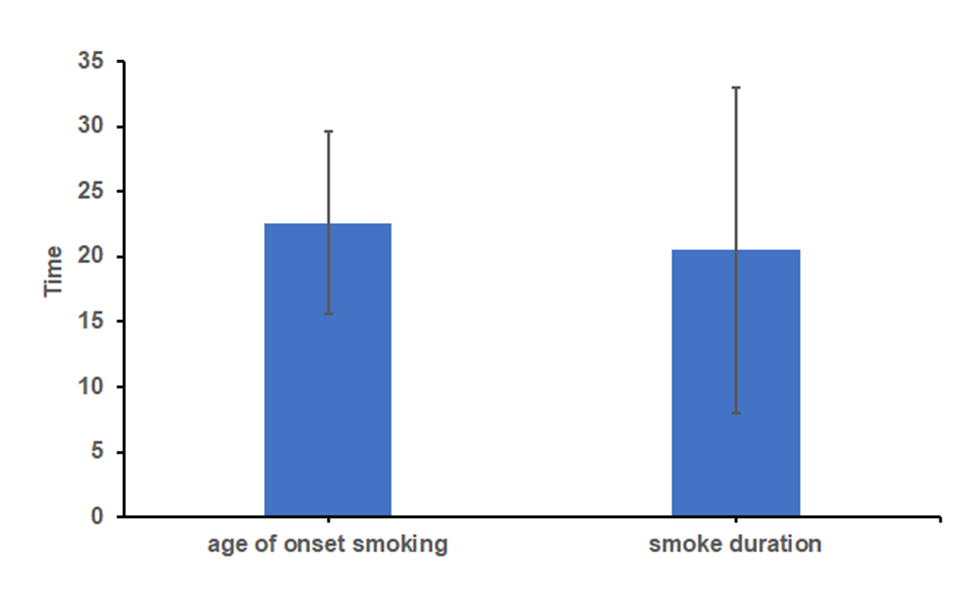

Supplement: S3 Fig — (TIF) [file pone.0225435.s005.tif]
